# Determinations of the peroxidative susceptibilities of cod liver oils by a newly-developed ^1^H NMR-based method: resistance of an antioxidant-fortified product isolated from pre-fermented sources

**DOI:** 10.1186/s13104-020-4932-6

**Published:** 2020-02-12

**Authors:** B. C. Percival, R. Zbasnik, V. Schlegel, M. Edgar, J. Zhang, M. Grootveld

**Affiliations:** 1 Leicester School of Pharmacy, De Montfort University, The Gateway, Leicester, LE1 9BH UK; 2 Natural Product Analysis Laboratory, Department of Food Science and Technology, University of Nebraska-Lincoln, 1901 N 21st Street, Lincoln, NE 68588-6205 USA; 3 Department of Chemistry, University of Loughborough, Epinal Way, Loughborough, LE11 3TU UK; 4 Green Pasture Products, 416 E. Fremont Street, O’Neill, NE 68763 USA

**Keywords:** Cod liver oil, Fermented cod liver oil, ^1^H NMR analysis, Lipid peroxidation, Aldehydes, Antioxidants, Hydrolytic collagenous degradation products, Biogenic amines, Ammonia, Oxygen radical absorbance capacity (ORAC)

## Abstract

**Objective:**

To explore the molecular composition and antioxidant status of four natural (unrefined) cod liver oil (CLO) products, three of which (Products 1–3) were non-fermented, whilst one (Product 4) was isolated from pre-fermented cod livers, and hence was naturally antioxidant-fortified. Potential antioxidants and aldehyde-scavenging agents were determined by recommended and/or ^1^H NMR methods; peroxyl radical-specific oxygen radical absorbance capacity (ORAC) values were measured fluorimetrically. The activities of such antioxidants were also investigated by assessing the susceptibilities/resistivities of these CLOs to thermo-oxidation by ^1^H NMR analysis, which monitored the time-dependent evolution of aldehydic lipid oxidation products at 180 °C.

**Results:**

Product 4 displayed much higher, albeit variable ORAC values (mean ± SEM 91.4 ± 19.5 mmol. trolox equivalents/kg) than those of Products 1–3, an observation arising from significant levels of peroxidation-blocking and/or aldehyde-consuming collagenous polypeptides/peptides, flavonones, biogenic amines, total phenolics, tannins, and ammoniacal agents therein. All of these agents were undetectable in Products 1–3. Quantitative considerations indicated that collagenous gel agents (present at ca. 1.5% (w/w)) were the most powerful Product 4 antioxidants. Significantly lower levels of aldehydes were generated in this product when exposed to thermal-stressing episodes. Results confirmed the enhanced peroxidative resistivity of a pre-fermented, antioxidant-rich natural CLO over those of corresponding non-fermented products. Product 4: Green Pasture Blue Ice™ fermented cod liver oil.

## Introduction

The fermentation of fish food sources with lactate-generating and salt-tolerant bacteria generates a multitude of valuable products, e.g. key biomolecules with protective microbicidal and/or antioxidant functions. Recent developments in the fermented food research area have been thoroughly reviewed in [1]. Boosting of the total antioxidant capacities of foods, following their exposure to fermenting microbes arises, from the liberation of lipid peroxidation chain-terminating antioxidants from the enzymatic breakdown of a wide range of high-molecular-mass biomolecules present. Since marine oils generated from the fermentation of fish (e.g. cod) livers serves as an example of fermented supplemental foods, it is conceivable that a significant and prolonged antioxidant fortification is naturally achievable therein via this production strategy.

Therefore, we employed high-resolution ^1^H NMR, and a range of additional analytical techniques, in order to fully evaluate the molecular compositions and antioxidant potential of four ω-3 polyunsaturated fatty acid (PUFA)-rich, commercially-available, natural (unrefined) cod liver oil (CLO) products, specifically one produced from a fermented cod liver source (and henceforth termed ‘fermented CLO’, Product 4), and three from corresponding unfermented matrices (Products 1–3).

Moreover, the peroxidative resistivities of these products on exposure to sequential thermal stressing episodes (TSEs) was explored by an NMR-based thermo-oxidation resistivity assay (abbreviated TORA). This method involved the exposure of oils to TSEs at 180 °C for durations of 0–90 min., coupled with the time-dependent simultaneous monitoring of thermo-oxidative changes in the concentrations of a series of secondary aldehydic lipid oxidation products (LOPs), natural acylglycerols and antioxidants, for example. This method may serve as a valuable supporting alternative for the commonly-employed high-temperature, accelerated Rancimat^®^ method [2] available for such assessments. Indeed, it offers a very high level of selectivity.

## Main text

### Methods

Different batches of Products 1–4 (n = 4 or 5) were purchased from commercial US retail outlets. CLO FA contents were determined by a ^1^H NMR method [3] (Table 1), and also by AOAC Method 996.06 (Additional file 1: section S1) [4]. Results acquired demonstrated a high level of analytical agreement between these methods. NMR analysis at an operating frequency of 600 MHz, and CLO sample preparations for this purpose, were performed as described in Additional file 1: section S2.

**Table 1:** ^1^H NMR-determined mean ± SEM molar% FA contents of Products 1–4, along with those for their [DHA]:[EPA] molecular concentration ratios

Product	Total ω-3 FAs (molar%)	EPA (molar%)	DHA (molar%)	UFAs (molar%)	SFAs (molar%)	[DHA]:[EPA]
1	21.5 ± 0.23	9.7 ± 0.23	10.4 ± 0.10	80.6 ± 0.13	19.4 ± 0.13	1.06 ± 0.02
2	25.8 ± 0.02	12.0 ± 0.07	12.3 ± 0.01	82.0 ± 0.10	18.0 ± 0.10	1.03 ± 0.003
3	25.3 ± 0.12	12.2 ± 0.08	12.3 ± 0.01	81.8 ± 0.08	18.1 ± 0.08	1.02 ± 0.003
4	27.0 ± 0.15	15.5 ± 0.02	9.3 ± 0.01	83.4 ± 0.06	16.6 ± 0.06	0.60 ± 0.001

A total of n = 4 repeat determinations were performed for each CLO product tested

*DHA* docosahexaeneoic acid, *EPA* eicosapentaeneoic acid

Biogenic amines (BAs), ammonia, retinol, flavonones, tannins, and further antioxidants and nutrients such as α-tocopherol, etc. were determined in these products by recommended HPLC, LC–MS/MS or spectrophotometric methods (Additional file 1: sections S3 and S4). Determinations of collagenous antioxidants and their molecular mass ranges were performed by HPLC and SDS-PAGE analyses respectively (Additional file 1: section S5). Oxygen radical absorbance capacity (ORAC) indices of Products 1–4 were determined as in [5] (Additional file 1: section S6). SEM values reported correspond to ‘between-batch’ ones for each product tested, i.e. n = 4 different batches for Products 1–3, and n = 5 batches for Product 4.

Products 1–4 underwent TSEs at 180 °C according to the description in section S2.1, and outlines of the ^1^H NMR analysis of aldehydic LOPs and the preprocessing of data acquired therefrom, are provided in Additional file 1: sections S2.2 and S2.3 respectively. ^1^H NMR-determined aldehyde concentrations were FA-normalised.

Details concerning the design of the work-tasks performed, and the statistical analyses of all datasets acquired (including an ANCOVA model for the TORA TSE experiments), are outlined in Additional file 1: section S7.

### Results

^1^H NMR analysis and alternative supporting analytical techniques revealed that Product 4 contained significant levels of peroxidation-blocking antioxidant fermentation products, including BAs such as 2-phenylethylamine and tyramine (total reactive amine functions 1.39 ± 0.35 mmol./kg (mean ± SEM)), total flavonones (896 ± 199 mg/kg), total phenolics (80 ± 4 mg/kg) and tannins (112 ± 67 mg/kg), and also unexpectedly high concentrations of collagen and its hydrolytic degradation products (total 1.5% (w/w) of 1.6% (w/w) total protein content), together with ammonia (80 mg/kg), another fermentation product. All these antioxidants were undetectable in Products 1–3. Total carotenoid levels in Product 4 (1.86 ± 0.91 mg/kg) were significantly higher than those of all other products (*p *< 0.01). However, α-TOH and retinol concentrations (0.13 ± 0.04 and 0.10 ± 0.03 mmol./kg in Product 4 respectively) were found not to be significantly different from those of Products 1–3.

The molecular mass range of Product 4 collagenous species detectable was found to be highly variable (i.e. 5–270 kDa), data confirming that much of the collagen was present as its hydrolytic degradation products (CDPs) generated during cod liver fermentation.

Determination of ORAC values, which provide measures of the abilities of edible oil products to self-protect their PUFAs against oxidative damage and henceforth rancidity, confirmed that fermented Product 4 had a substantially higher peroxyl radical-scavenging capacity than those of unfermented Products 1–3, i.e. mean ± SEM value 91 ± 19 versus 5 ± 1, 46 ± 5 and 6 ± 1 mmol. trolox equivalents/kg for Products 1, 2 and 3 respectively (*p *< 10^−4^).

**Fig. 1:**
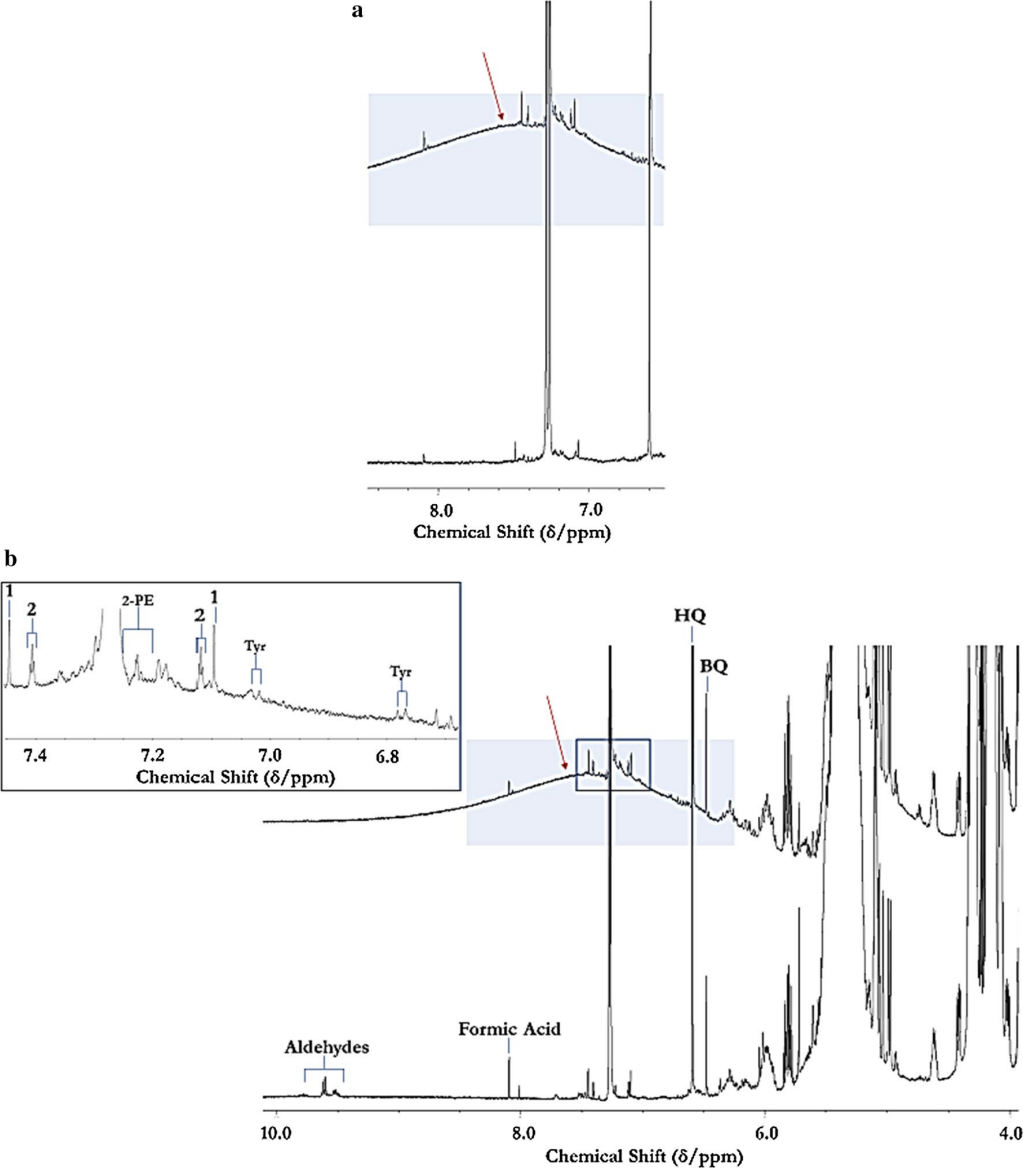
**a** Expanded 6.50–8.50 ppm region of 600 MHz ^1^H NMR profiles acquired on Products 4 (top) and 3 (bottom), showing the unusual, very broad resonance detectable in that of Product 4 only; **b** Expanded 6.50–8.50 ppm region of the 600 MHz ^1^H NMR spectrum of Product 4 in C^2^HCl_3_ solution before (top) and after (bottom) treatment with a 10 µL volume of ^2^H_2_O. HQ and BQ, singlet resonances assignable to the aromatic ring protons of 2,5-di-*tert*-butylhydroquinone (DTBHQ) and its benzoquinone oxidation product respectively, the latter arising from the former’s ability to block peroxidation of CLO UFAs during periods of laboratory storage and sample preparation (the intensity ratio of these two signals therefore serves as a measure of such artefactual peroxidation taking place during such periods). Inset abbreviations: 2-PE and Tyr, aromatic ring protons of 2-phenylethylamine and tyramine respectively; 1 and 2, ^13^C satellites of the residual CHCl_3_ solvent and 1,3,5-trichlorobenzene standard ^1^H NMR resonances respectively

Multicomponent ^1^H NMR analysis was therefore further employed to explore the nutrient and antioxidant status of Product 4. A very broad signal present in the ^1^H NMR profiles of all batches of Product 4 samples analysed, but not in those of the non-fermented Products 1–3, provided further valuable evidence for the availability of CDPs in the former, along with possible contributions from ammonia (Fig. 1), the latter conceivably as ion-pair complexes of NH_4_^+^ with free FA anions; chemical model system experiments confirmed this (data not shown). As expected, this resonance was completely ^2^H_2_O-extinguishable (Fig. 1b), and was also eliminated from spectra following short duration (10–20 min.) TSEs. Application of the Carr–Purcell–Meiboom–Gill (CPMG) spin-echo pulse sequence generated ^1^H NMR profiles without this signal, an observation also consistent with its CDP and/or ammoniacal identities. Electronic integrations of this resonance provided an estimated mean single proton-equivalent concentration value of approximately 400 mmol./kg. The total protein content of Products 1–3 was reported as none detectable, i.e. below the reporting limit. The water content of Product 4 varied from 0.3–1.0% (w/w).

**Fig. 2:**
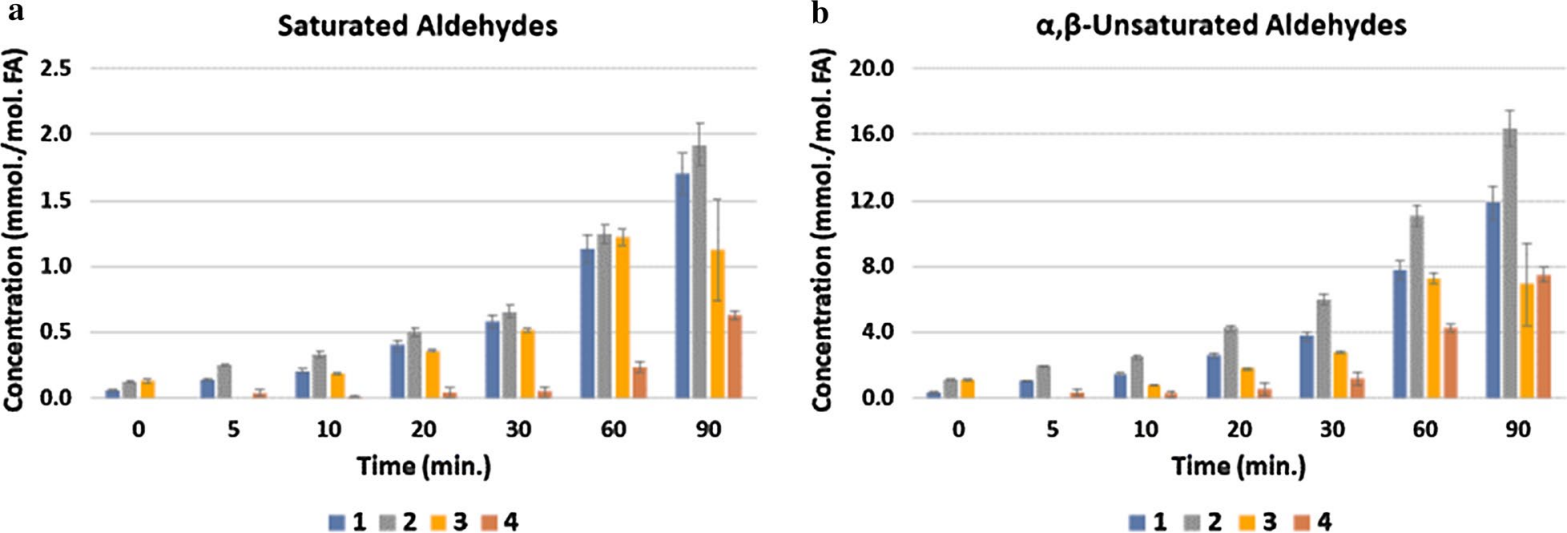
Mean ± SEM concentrations of **a** total saturated, and **b** total α,β-unsaturated secondary aldehydic LOPs (mmol./mol. FA) determined in CLO Products 1–4 at the 0, 5, 10, 20 30, 60 and 90 min. sampling time-points when exposed to TORA TSEs conducted at 180 °C. The colour codes beneath the abscissa axes correspond to Products 1, 2, 3 and 4

TORA experiments revealed that all aldehyde concentrations monitored increased with increasing TSE duration for each CLO product investigated (Additional file 1: Figures S1 and Fig. 2). Significantly lower levels of cytotoxic and genotoxic secondary aldehydic LOPs (both total saturated and α,β-unsaturated classes) were formed in Product 4 than those found in the non-fermented ones when exposed to these TSEs (*p *< 10^−6^ for both forms).

### Discussion

This study demonstrated that Product 4 PUFAs were more resistant to thermally-induced peroxidative damage than those of the non-fermented, albeit unrefined products when monitored by the time-dependent generation of secondary aldehydic LOPs during TSEs. These observations are explicable by the high levels of fermentation-sourced antioxidants, and/or agents with the ability to directly consume aldehydes and/or their precursors (including CDPs and ammonia), found in Product 4.

In principle, all Product 4 antioxidants monitored here have the ability to (1) suppress the thermally-stimulated, self-propagating peroxidation of CLO PUFAs, especially for the highly-susceptible ω-3 FAs DHA and EPA (including CDPs, ammonia and BAs, the latter with PUFA-derived pentadienyl carbon-centred radical scavenging capacity [6]); (2) attenuate the fragmentation of PUFA-derived hydroperoxides to toxic aldehydes (potentially many CLO electron-donors); and/or (3) directly chemically scavenge aldehydic LOPs (CDPs, ammonia, BAs). However, the activities of these protectants in these processes are highly dependent on their available CLO concentrations, and the thermodynamic favourabilities and rates of each LOP–neutralising reaction involved.

Notwithstanding, the above series of phenolic antioxidants, BAs, flavonones and tannins, etc. found in Product 4 cannot possibly be responsible for its extremely high ORAC value, since their maximal total (summed) concentration (estimated to be ≤ 8 mmol./kg) is markedly lower than its total peroxyl radical-trapping capacity, i.e. it is ca. 12-fold less than its high mean ORAC value of 91 trolox equivalents/kg (weight-equivalent ORAC values determined on sage, peppermint, oregano and fresh basil are 320, 140, 140 and 44 trolox equivalents/kg [7]). Moreover, concentrations of α-TOH and mean total carotenoids available in all products evaluated (0.7–1.9 mg/kg for the latter) are clearly insufficient to effectively compete for lipid alkoxyl and peroxyl radical reactants generated in TSE-stimulated CLO products exposed to TSEs. Hence, it appears that the major contributor towards this highly elevated index is the total protein/peptide content of this CLO, which is predominantly accountable by collagen and its CDPs.

It is also notable that toxicological ramifications concerning the human ingestion of aldehydic LOPs generated are of much public health significance, i.e. highly relevant to those consuming bottled and/or supplemental ω-3 FA-rich marine oil products as a health benefit, but which may be inadvertently contaminated with such toxins through their exposure to excessive periods of heat, light and peroxidation-promoting atmospheric O_2_. The detection of secondary aldehydic LOP toxins in some (unheated) CLO products (e.g., Figures 2 and Additional file 1: S1) may adversely negate the potential positive health benefits putatively offered by their high ω-3 FA contents [8].

Intriguingly, natural, untampered fortification of such products with a series of powerful chain-breaking antioxidants and/or CDP adducts through fermentation processes appears to exert substantive protective effects. Indeed, collagen-rich fish scale extracts have been demonstrated to exert an extremely efficacious lipid radical-trapping antioxidant activity [9]. In view of these vigorous properties, Product 4 is anticipated to have an extended shelf-life over those of products with lower or much lower ORAC values; experiments to further explore this are therefore required. The availability of a diverse range of amine functions in the fermented product is of further importance, since these may directly neutralise aldehyde toxins via Schiff base and/or Michael addition reactions.

However, the mean [DHA]:[EPA] concentration ratios of each CLO product investigated (Table 1) revealed that Product 4’s value was only ca. 60% of those of all of the other products studied, and this significantly lowered value may also be partially responsible for its enhanced resistivity to TORA TSE challenges. Notably, the relative peroxidative susceptibilities of DHA:EPA is in a 4:3 ratio [10].

Results acquired in this investigation are also relevant to the peroxidation of ω-3 FAs naturally present in fish food products when exposed to shallow-frying episodes at similar temperatures to that of our TSEs, usually for periods of up to 12 min.

The NMR-based TORA strategy developed herein serves as a highly versatile and valuable technique for evaluations of the peroxidative resistivities/sensitivities of CLO products, and edible oils in general. Indeed, this NMR analysis approach may offer advantages over the established, heat-dependent Rancimat^®^ method [2] for such evaluations, i.e. its ability to provide valuable information on the molecular nature and levels of many LOP classes (e.g., > 11 classes of aldehydic fragments). In principle, such detailed observations regarding the identities, time-dependence and levels of LOPs generated during TSEs are likely to engender the provision of valuable clues regarding their CLO UFA sources, and mechanisms for their peroxidative evolution: for example, the production of acrolein, propanal and malondialdehyde from ω-3 FAs (along with oxidation of triacylglycerol glycerol backbones for the former), and alka-2,4-dienals, 4,5-diepoxy-, 4-hydroxy- and 4-hydroperoxy-*trans*-2-alkenals from PUFAs in general, and not MUFAs. To the best of our knowledge, this is the first time that such a multi-analytical lipidomic ^1^H NMR analysis strategy has been employed to explore the peroxidative resistivities/sensitivities of CLO products.

## Limitations

One limitation of the present study is that the abilities of chain-breaking and/or alternative activity antioxidants, and aldehyde-scavenging amines, to exert their protective functions towards CLO UFAs at high TSE temperatures may be constrained, either because of their volatilities (e.g., selected BAs) and/or their thermal instabilities (e.g., tocopherols, carotenoids and presumably CDPs). However, such protective actions are expected to be much more readily exerted at ambient or lower storage temperatures, and hence will be prolonged and molecularly-extensive. However, elevated TSE temperatures will markedly enhance the rates, and possibly also thermodynamic favourabilities, of the reactions involved.

A further limitation is the volatility of selected aldehydic LOPs, i.e. *trans*-2-heptenal, *trans,trans*-2,4-decadienal and *n*-hexanal from linoleoylglycerol peroxidation all have boiling points (b.pts) below the TSE temperature employed (180 °C), and acrolein and propanal from linolenoylglycerol peroxidation have b.pts of only 53 and 49 °C respectively. Hence, the concentrations of all aldehyde classes monitored in each CLO product represent only the fraction of those remaining in the oil medium at specified TORA sampling time-points. This knowledge is of paramount public health concern in view of the inhalation of aldehyde toxin-containing cooking fumes by humans during frying practices employing PUFA-rich frying oils, e.g. workers employed in restaurant outlets with poor or inadequate ventilation precautions, or those engaged in wok cooking practices.

## Supplementary information

**Additional file 1. Section S1.** Method for gas chromatographic (GC) determinations of CLO fatty acid (FA) contents. **Section S2.** TORA TSEs and ^1^H NMR sample preparations and analysis. **Section S3.** Analysis of CLO products for total phenolics, flavonoids, flavonones, anthocyanins, tannins, and carotenoids and chlorophylls. **Section S4.** Biogenic amine analysis. **Section S5.** Analysis of CLO product protein, collagen, ammonia and moisture. **Section S6.** ORAC value determinations performed on CLO products. **Section S7.** Experimental design and statistical analysis of datasets acquired. **Section S8.** Additional file references. **Figure S1.** Expanded aldehydic-CHO proton (9.4-10.0 ppm) regions of the 600 MHz ^1^H NMR spectra of CLO products exposed to TORA TSEs at 180 °C for periods of 0, 30 and 90 min.

## Data Availability

Datasets employed and analysed in this investigation are available from the corresponding author on reasonable request.

## Abbreviations

ANCOVA: Analysis-of-covariance
BA: Biogenic amines
CDP: Collagen degradation product
CLO: Cod liver oil
CPMG: Carr–Purcell–Meiboom–Gill
DHA: Docosahexaeneoic acid
DTBHQ: 2,5-di-*tert*-butylhydroquinone
EPA: Eicosapetaeneoic acid
FCLO: Fermented cod liver oil
FA: Fatty acid
HPLC: High- performance liquid chromatography
LC–MS/MS: Liquid chromatography/mass spectrometry/mass spectrometry
LOP: Lipid oxidation product
MUFA: Monounsaturated fatty acid
NMR: Nuclear magnetic resonance
ORAC: Oxygen radical absorbance capacity
PUFA: Polyunsaturated fatty acid
SDS-PAGE: Sodium dodecyl sulphate–polyacrylamide gel electrophoresis
SEM: Standard error of the mean
SFA: Saturated fatty acid
TORA: Thermo-oxidation resistivity assay
TSE: Thermal stressing episode
UFA: Unsaturated fatty acid
α-TOH: α-tocopherol
