# Supplementary material for: Determinations of the peroxidative susceptibilities of cod liver oils by a newly-developed 1H NMR-based method: resistance of an antioxidant-fortified product isolated from pre-fermented sources
Source: BMC Res Notes. 2020 Feb 12;13:73. doi: 10.1186/s13104-020-4932-6 (PMC7017492; doi:10.1186/s13104-020-4932-6)
Supplement: Supplementary file 1 — Additional file 1. Section S1. Method for gas chromatographic (GC) determinations of CLO fatty acid (FA) contents. Section S2. TORA TSEs and 1H NMR sample preparations and analysis. Section S3. Analysis of CLO products for total phenolics, flavonoids, flavonones, anthocyanins, tannins, and carotenoids and chlorophylls. Section S4. Biogenic amine analysis. Section S5. Analysis of CLO product protein, collagen, ammonia and moisture. Section S6. ORAC value determinations performed on CLO products. Section S7. Experimental design and statistical analysis of datasets acquired. Section S8. Additional file references. Figure S1. Expanded aldehydic-CHO proton (9.4-10.0 ppm) regions of the 600 MHz 1H NMR spectra of CLO products exposed to TORA TSEs at 180 °C for periods of 0, 30 and 90 min. [file 13104_2020_4932_MOESM1_ESM.docx]

**Determinations of the Peroxidative Susceptibilities of Cod Liver Oils by a Newly-Developed ^1^H NMR-Based Method: Resistance of an Antioxidant-Fortified Product Isolated from Pre-Fermented Sources**

**B. C. Percival^1^, R. Zbasnik^2^, V. Schlegel^2^, M. Edgar^3^, J. Zhang^4^ and M. Grootveld^1*^**

**^1^Leicester School of Pharmacy, De Montfort University, The Gateway, Leicester LE1 9BH, United Kingdom.**

**^2^Natural Product Analysis Laboratory, Department of Food Science and Technology, University of Nebraska-Lincoln, 1901 N 21st Street, Lincoln, NE 68588-6205, USA.**

**^3^Department of Chemistry, University of Loughborough,** **Epinal Way, Loughborough LE11 3TU, United Kingdom.**

**^4^Green Pasture Products, 416 E. Fremont Street, O’Neill, NE 68763, USA.**

**ADDITIONAL FILE SECTION**

***Author for Correspondence: Professor Martin Grootveld, Leicester School of Pharmacy, De Montfort University, The Gateway, Leicester LE1 9BH, United Kingdom.**

**Tel.: +44-(0)116-250-6443**

**Email: mgrootveld@dmu.ac.uk**

**Additional File Contents**

**Section S1: Method for Gas Chromatographic (GC) Determinations of CLO Fatty Acid (FA) Contents**

**Section S2: TORA TSEs and ^1^H NMR Sample Preparations and Analysis**

**Section S3: Analysis of CLO Products for Total Phenolics, Flavonoids, Flavonones, Anthocyanins, Tannins, and Carotenoids and Chlorophylls**

**Section S4: Biogenic Amine Analysis**

**Section S5: Analysis of CLO Product Protein, Collagen, Ammonia and Moisture**

**Section S6: ORAC Value Determinations Performed on CLO Products**

**Section S7: Experimental Design and Statistical Analysis of Datasets Acquired**

**Section S8: Additional File References**

**Figure S1.** Expanded aldehydic-CHO proton (9.4-10.0 ppm) regions of the 600 MHz ^1^H NMR spectra of CLO products exposed to TORA TSEs at 180^o^C for periods of 0, 30 and 90 min.

**Section S1: Method for Gas Chromatographic (GC) Determinations of CLO Fatty Acid (FA) Contents**

CLO FA contents were determined by AOAC Method 996.06, which involves capillary GC-flame ionization detector (FID) analysis of fatty acid methyl esters (FAMEs) [4].

Accurately weighed samples of CLOs (100-200 mg) were dissolved in a solution of chloroform (2.00 mL) which contained the internal standard triundecanoin (5.00 mg/mL). Pyrogallol (100 mg) and ethanol (2.00 mL) were then added and the mixture rotamixed prior to adding a solution of 8.3 mol./L hydrochloric acid (10.0 mL), and then allowing to equilibrate at a temperature of 75ºC for 40 min. with shaking at 10 min. intervals. Subsequently, lipids were extracted with 25 mL of diethyl ether and 25 mL hexane. The solvent was removed under N_2_, re-collected and then added to a 5 mL volume Reacti-Vial containing 4.0 mL of a 50:50 (v/v) mixture of diethyl ether:chloroform. This solvent system was removed under N_2_, and then 1.0 mL of toluene and 1.0 ml of 14% (w/v) solution of boron triflouride in methanol were added prior to tight capping and incubating at 90ºC for a period of 40 min. Samples were allowed to cool to ambient temperature, and then were treated with 4.0 mL of doubly-distilled water, 1.0 g of anhydrous sodium sulphate and a 1.0 mL volume of hexane. Samples were then centrifuged to separate the phases, and the organic (hexane) one was collected for injection purposes.

FAs were separated and identified using an Agilent 7820A GC system coupled with a flame ionisation detector and an Agilent G4567A autosampler. 1.0 µL volumes of prepared samples were injected onto a Suppelco RT-2560 column. The injection port and detector were maintained at 225ºC and 250ºC respectively, with the oven starting at 100ºC for 4 min. prior to increasing to 240ºC at a 3ºC/min. rate, and then maintaining at this temperature for 20 min. The retention times of FAs were determined by standard injection, and their total levels were computed from expression of their GC peak areas to that of the triundacanoin internal standard.

**Section S2: TORA TSEs and ^1^H NMR Sample Preparations and Analysis**

**S2.1 *Exposure of CLO samples to TORA thermal-stressing episodes (TSEs), and their preparation for ^1^H NMR analysis***

Experiments were conducted by a ‘blinded’ laboratory researcher. Each 90 min. heating cycle was completed n = 3 replicated sessions for all CLO products investigated. TSEs featured the heating of 6.00 mL volumes of CLO samples in air-dried 250 ml volume glass beakers fitted and clamped within a thermostatted silicon oil bath, and maintained at a temperature of 180±2^o^C during the total heating period. Samples (0.20 ml) of oil samples were obtained at the 0, 10, 20, 30, 60 and 90 min. heating time-points for ^1^H NMR analysis. Following collection, samples were immediately treated with 0.20 mL of a 50.0 mmol./L solution of 2,5-di-*tert*-butylhydroquinone (DTBHQ) in C^2^HCl_3_ in order to prevent the artefactual generation of LOPs during episodes of storage and sample preparation (final added concentration 6.58 mmol./L). Pending analysis, samples were stored in tight-sealed containers within a darkened zone in a freezer at -80^o^C.

**S2.2 *^1^H NMR analysis***

^1^H NMR measurements on the above samples were conducted on a 600 MHz Bruker Avance AV-600 or Jeol JNM-ECZR 600 spectrometers (Kingston University London and Leicester School of Pharmacy facilities respectively) operating at frequencies of 600.13 and 600.17 MHz respectively, and a probe temperature of 293 K. Aliquots (0.30 mL) of the resulting 2:1 (v/v) CLO:DTBHQ solution admixtures were then treated with 0.40 ml of deuterated chloroform (C^2^HCl_3_) containing 0.34 mol./L tetramethylsilane (TMS), and 0.060 ml of 66.00 mmol./L 1,3,5-trichlorobenzene (1,3,5-TCB): the C^2^HCl_3_ diluent provided a field frequency lock, both TMS and 1,3,5-TCB acted as internal chemical shift references (δ = 0.00 and 7.20 ppm respectively).

These solutions were then placed in 5-mm diameter NMR tubes. Typical pulsing conditions were: zg30 pulse program, with 2 dummy scans; 128 free induction decays (FIDs), each involving 65,536 data points; an acquisition time of 2.66 s; a sweep width of 20.55 ppm. ^1^H NMR resonances were routinely assigned by considering of chemical shift values, coupling patterns and coupling constants. One- and two-dimensional COSY and TOCSY spectra were acquired to confirm ^1^H NMR assignments as previously described [S1-S3].

Carr-Purcell-Meiboom-Gill (CPMG) spectra [S4,S5] of Product 4 were obtained on a JEOL-ECZ-500 500 MHz spectrometer (University of Loughborough facility) which used Delta version 5.2.1 software. A spectral width of 16.0 ppm with a precisely-set 1H P90 of 7.6 µs at 18 dB attenuation was employed, and 64 scans were acquired. The τ delay was 0.9924 ms, and 500 loops were cycled.

.^1^H NMR spectra of CLO samples treated with a 10 μL volume of amide function proton-extinguishing ^2^H_2_O were obtained on the JEOL-ECZR 600 spectrometer. Typical acquisition parameters were: acquisition time 1.45 s; 16 scans; receiver gain of 26; 45^o^ pulse angle; 3.315 µs pulse; repetition time 6.45 s; 16,384 datapoints acquired; and 9,000 Hz spectral width. Another strategic approach involved exclusion of the above 1,3,5-TCB internal standard to remove interferences from its ^13^C satellite resonances with the observation of those of Product 4’s aromatic antioxidants.

**S2.3 *Preprocessing of ^1^H NMR spectral profiles: Determinations of different classes of aldehydic LOPs in CLOs***

Preprocessing of the aldehydic LOP regions of ^1^H NMR profiles was conducted by using of a separate macro for the ‘intelligent bucketing’ processing sub-routine. These were performed using the ACD/Labs Spectrus Processor 2019 software package (ACD/Labs, Toronto, Ontario, Canada M5C 1T4), which gave rise to a CLO dataset matrix consisting of intelligently-selected buckets (ISBs) relating to the-CHO function signals of different classes of aldehydes, specifically *d*, δ = 9.49-9.51 ppm (*trans*-2-alkenals); *d*s, δ = 9.51-9.55 and 9.60-9.63 ppm (*trans,trans-* and *cis,trans-*alka-2,4-dienals respectively); *d*, δ = 9.55-9.57 ppm (4,5-epoxy-*trans*-2-alkenals/acrolein); *t*, δ = 9.74-9.76 ppm (*n*-alkanals); *t*, 9.76-9.78 ppm (4-oxo-*n*-alkanals); and *t*, 9.79-9.80 ppm (low-molecular-mass *n*-alkanals such as ethanal, *n*-propanal and *n*-butanal), an adaption of the approach previously described in [S6]. Additional aldehyde-CHO group resonances were also observed, and two quantifiable ones were within the δ = 9.78-9.79 and 9.80-9.82 ppm ISB regions (both multiplets, Figure S1).

The intensities of resonances corresponding to each of the above aldehyde-CHO function ISBs were normalised to that which incorporated all acylglycerol-terminal-CH_3_ function ones (δ = 0.82-0.99 ppm), and after allowing for the relative numbers of ^1^H nuclei contributing towards these signals, CLO sample aldehyde concentrations were expressed as µmol. or mmol. per mol. of total FAs. Total saturated aldehyde concentrations were estimated by primarily summing the δ = 9.74-9.76 and 9.79-9.80 ppm ISBs, and total α,β-unsaturated aldehydes were estimated by summing the δ = 9.49-9.51, 9,51-9.55, 9,55-9.57, 9.60-9.63 and 9.76-9.78 ppm ISBs.

‘Between-replicate’ sample coefficients of variation for all aldehyde class determinations ranged from 4 to 12% for all CLO products investigated; that for repeated ^1^H NMR determinations made on exactly the same oil sample were < 3%.

Linear calibration curves for typical *trans*-2-alkenals and *n*-alkanals (0-500 µmol./L^-1^ and 0.50-30.00 mmol./L concentration ranges) had R^2^ values ≥ 0.995 for neat C^2^HCl_3_ solutions, and ≥ 0.984 for aldehyde-treated (C^2^HCl_3_-diluted) CLO samples prepared in the above manner.

**Section S3: Analysis of CLO Products for Total Phenolics, Flavonoids, Flavonones, Anthocyanins, Tannins, and Carotenoids and Chlorophylls**

Total phenolics, flavonoids and flavones were determined by recommended spectrophotometric methods, the latter involving 2,4-dinitrophenylhydrazine as a derivatising reagent [SS7/S8, S9 and S10 respectively]. Anthocyanins and tannins were also determined spectrophotometrically [S11 and S12 respectively], the former via a pH-differential approach. Retinol and tocopherol levels were simultaneously monitored by a HPLC method [S13], whereas carotenes and chlorophylls were determined by an established spectrophotometric approach [S14].

**Section S4: Biogenic Amine Analysis**

Simultaneous LC/MS/MS analysis of biogenic amines (11 in total) involved the filtering of pre-specified aliquots of CLO samples utilising a 0.45-μm filter paper, shaking with a 20 mL volume of 70% (v/v) methanol/30% (v/v) water for a 20 min. period, and then centrifuging at 7,000 r.p.m. at 4^o^C for 20 min. The total volume of the clear supernatant was then transferred to a 1.7 mL amber auto-sampler vial for LC/MS/MS analysis.

A Dionex UltiMate® 3000 Rapid Separation LC (RSLC) analytical system comprising a pump, vacuum degasser, auto-sampler, column compartment, and a variable wavelength electronic absorption detection system was utilised, together with an Applied Biosystems/MDS Sciex API 3200TM mass spectrometer.

In order to enhance analytical sensitivity, acetonitrile was added to the LC eluent at a rate of 0.40 mL/min. at the Turbo VTM source’s “tee” piece, and this promoted the generation of dry ions during the early part of the sequential time program, during which the water content was high. A 3-μm 50 x 2.1 mm Pinnacle® DB PFPP column was employed for this analysis, since this selection was found to be optimal for biogenic amine separations, rapid analysis and robustness. Mobile phase A comprised water containing either 0.05 or 0.10% (w/v) of the ion-pair reagent trifluoroacetic acid (TFA), and mobile phase B consisted of acetonitrile containing an equivalent TFA concentration.

For the MS/MS detection system, eluting analytes were monitored in positive ion, multiple-reaction monitoring (MRM) mode.

**Section S5: Analysis of CLO Product Protein, Collagen, Ammonia and Moisture**

CLO protein contents were determined by the combustion method MWL F0 014 S10 [S15], ammonia by the SM 4500-NH3 C-(1997) method, and moisture by the AOCS Official Method Ca 2a-45 [S16], for which the standard minimum reporting level was 0.1% (w/w).

The molecular mass and contents of collagen in Product 4 were investigated by two approaches. Firstly, SDS-PAGE analysis was performed: this involved the preliminary precipitation of proteins and a collagen extraction process, followed by SDS-PAGE analysis, readings and their interpretation, in order to determine the molecular mass range and distribution of collagenous agents in this product. Secondly, collagen levels were determined as hydroxyproline equivalents by an HPLC method featuring phenylisothiocyanate (Edman’s reagent) as a derivatising agent, a C18 column and a spectrophotometric detection system set at a wavelength of 254 nm.

**Section S6: ORAC Value Determinations Performed on CLO Products**

An accurately weighed 10 to 15 µg quantity of oil was dispersed in 0.50 mL of 1:1 (v/v) acetone:water containing 7% (w/v) added methyl-β-cylcodextrin. Samples were then diluted 50-fold in 75 mmol./kg phosphate-buffered saline (PBS) at pH 7.40, and subsequently 25 µL volumes were transferred onto a 96-well plate in triplicate. A 150 µL volume of 0.30 nmol./L fluorescence solution was then added to each well, and their baseline fluorescence was monitored using excitation and emission wavelengths of 485 and 520 nm respectively. A 25 µL aliquot of a solution containing 800 mg of 2,2’-azobis(2-methylpropionamidine) dihydrochloride/mL in 75.0 mM PBS at pH 7.4 was added to each well to initiate the reaction, and then the fluorescence intensity was monitored at 1.5 min. intervals for a total 2.00 hr. duration. Time-dependent overall florescence intensities were compared with those of solutions of 6-hydroxy-2,5,7,8-tetramethylchoman-2-carboxylic acid (Trolox) solutions (0.80 to 100 µg/mL) in methanol as calibration standards [5].

**Section S7: Experimental Design and Statistical Analysis of Datasets Acquired**

The experimental design for univariate analysis of the total acylglycerol content-normalised ^1^H NMR aldehyde classification intelligently-selected bucket (ISB) intensity datasets (total saturated and α,β-unsaturated classes in this case) featured an analysis-of covariance (ANCOVA) model for determining the statistical significance of factors involved in the TORA TSE experiments. This model incorporated 2 prime factors, and a total of four sources of variation: (1) that 'between-CLO products’, a qualitative fixed effect (O*_a_*); (2) that ‘between-sampling time-points’, a ‘nested’ quantitative fixed effect (T*_(a)b_*); (3) the CLO product x heating time-point first-order interaction effect (OT*_ab_*); and (4) the ‘between-replicate’ effect, nested within factors (1) and (2) (R_(_*_ab_*_)_*_c_*). In this mathematical model (equation 1) y*_abcd_* represents the (univariate) aldehyde classification dependent variable values observed, μ its overall population mean value in the absence of any significant, influential sources of variation, and e*_abcd_* the unexplained error (residual) contribution.

*y_ijkl_* = μ + O*_a_* + T*_(a)b_* + OT*_ab_* + R_(_*_ab_*_)_*_c_* + e*_abcd_* (Eq. 1)

ANCOVA was performed using *XLSTAT2016* software (Addinsoft, Paris, France). *Post-hoc* analysis of significant effects observed between CLO products and sampling time-points were performed was conducted using Tukey’s test.

Further analysis of univariate aldehydic classification concentration data was performed by comparisons of their least square mean (LSM) values. For this purpose, LSM values for the ‘between-CLO product’ factor were determined by adjusting for the ‘between-heating sampling time-point’ variance contribution, and *vice-versa* for the latter. The statistical significance of these LSM differences were computed using Tukey’s *post-hoc* test.

Statistical analyses of differences observed between the mean antioxidant and nutrient contents of Products 1, 2, 3 and 4 were performed by ANOVA followed by Bonferroni post-hoc tests: mean values were computed from the values determined on n = 4 different batches of Products 1-3, and n = 5 different batches of Product 4. Mean±SEM values for these contents are available in the main text.

**Section S8: Additional File References**

[S1] Haywood RM, [Claxson](https://www.tandfonline.com/author/Claxson%2C+Andrew+W+D) AWD, [Hawkes](https://www.tandfonline.com/author/Hawkes%2C+Geoffrey+E) GE, [Richardson](https://www.tandfonline.com/author/Richardson%2C+David+P) DP,  [Naughton](https://www.tandfonline.com/author/Naughton%2C+Declan+P) DP, [Coumbarides](https://www.tandfonline.com/author/Coumbarides%2C+Gregory) G, [Hawkes](https://www.tandfonline.com/author/Hawkes%2C+Jane) J, [Lynch](https://www.tandfonline.com/author/Lynch%2C+Edward+J) EJ, [Grootveld](https://www.tandfonline.com/author/Grootveld%2C+Martin+C) MC. Detection of aldehydes and their conjugated hydroperoxydiene precursors in thermally-stressed culinary oils and fats: investigations using high resolution proton NMR spectroscopy. *Free Rad. Res.* 1995, 22:441-482.

[S2] Silwood CJL, Grootveld M. Application of high-resolution two-dimensional ^1^H and ^13^C nuclear magnetic resonance techniques to the characterization of lipid oxidation products in autoxidized linoleoyl/linolenoyglycerols. *Lipids* 1999, 34:741-756.

[S3] Claxson AWD, Hawkes GE, Richardson DP, Naughton DP, Haywood RM, Chander CL, Atherton M, Lynch EJ, Grootveld MC. Generation of lipid peroxidation products in culinary oils and fats during episodes of thermal stressing: a high field ^1^H NMR study. *FEBS Lett.* 1994, 355: 81-90.

[S4] Carr HY, Purcell EM. Effects of diffusion on free precession in nuclear magnetic resonance experiments. *Phys. Rev.* 1954, 94: 630-638.

[S5] Meiboom S, Gill D. Modified spin-echo method for measuring nuclear relaxation times. *Rev. Sci. Instrum.* 1958, 29, 688-691.

[S6] Moumtaz S, Percival BC, Parmar D, Grootveld KL, Jansson P, Grootveld M. Generation of toxic α,β-unsaturated and saturated aldehydes during simulated shallow frying episodes: comparisons of common frying oils with a novel high-stability algae oil product. *Sci. Rep.* 2019, 9, 4125:1-21.

[S7] Singleton VL, Rossi Jr. JA. Colorimetry of total phenolics with phosphomolybdic-phosphotungstic acid reagents. *Am. J. Enol. Vitic.* 1965, 16:144-158.

[S8] Adom KK, Liu RH. Antioxidant activity of grains. *J. Agri. Food Chem.* 2002, 50:6182-6187.

[S9] Popova M, Bankova V, Butovska D, Petkov V, Nikolova-Damyanova B, Sabatini AG, Marcazza GL, Bogdanov S. Validated methods for the quantification of biologically active constituents of poplar-type propolis. *Phytochem. Anal.* 2004, 15(4):235-240.

[S10] Shao Y, Xu F, Sun X, Bao J, Beta T. Phenolic acids, anthocyanins, and antioxidant capacity in rice (*Oryza sativa* L.) grains at four stages of development after flowering. *Food Chem.* 2004, 143: 90-96.

[S11] Shao Y, Xu F, Sun X, Bao J, Beta T. Phenolic acids, anthocyanins, and antioxidant capacity in rice (*Oryza sativa* L.) grains at four stages of development after flowering. *Food Chem.* 2004, 143: 90-96.

[S12] Bhat R, Sridhar K, Tomita-Yokotani K. Effect of ionizing radiation on antinutritional features of velvet bean seeds (*Mucuna pruriens*). *Food Chem.* 2007, 103:860-866.

[S13] Talwar D, Ha TKK, Cooney J. Brownlee C, St J O’Reilly DA. routine method for the simultaneous measurement of retinol, α-tocopherol and five carotenoids in human plasma by reverse phase HPLC. *Clinica Chimica Acta* 1998, 270:85-100.

[S14] Lichtenthaler HK, Bushmann, B. Chlorophylls and carotenoids: measurement and characterization by UV-VIS spectroscopy. *Current Protocols in Food Analytical Chemistry* 2001, p. F4.3.1-F4.3.8

[S15] AOCS Official Method Ca 2a-45 (Reapproved 2017): Moisture in Fats and Oils, Distillation Method.

[S16] King-Brink M, Sebranek, JG. Combustion method for determination of crude protein in meat and meat products: collaborative study. *J AOAC Int*. 1993, 76(4):787–793.

**Figure S1**

**(a)**


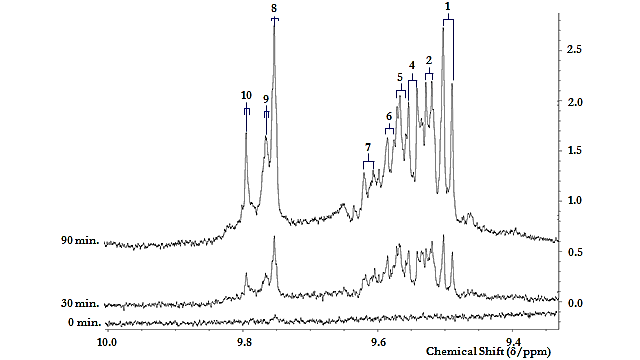
 **(b)**
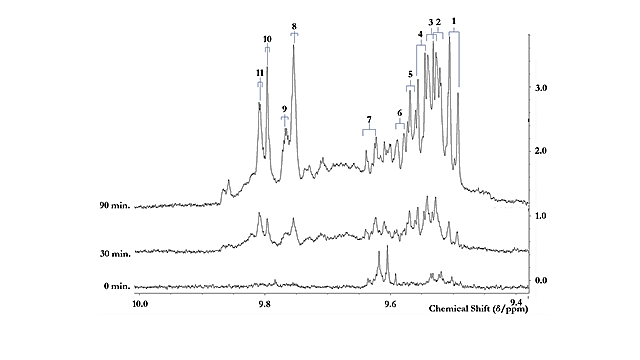


**Figure S1.** Expanded aldehydic-CHO proton (9.4-10.0 ppm) regions of the 600 MHz ^1^H NMR spectra of **(a)** CLO Product 4, and **(b)** CLO Product 3 exposed to TORA TSEs at 180^o^C for periods of 0 (bottom), 30 (middle) and 90 min. (top profile). Typical spectra are shown (the right-hand side ordinate axes indicate the relative intensities of profile resonances). Abbreviations: -CHO function resonances of 1, *trans*-2-alkenals; 2 and 3, 2 x molecular mass homologue classes of *trans*,*trans*-2,4-alkadienals; 4,4,5-epoxy-*trans*-2-alkenals; 5, acrolein; 6, combined 5-hydroxy-/5-hydroperoxy-*trans*-2-alkenals; 7, *cis,trans*-2,4-alkadienals; 8, *n*-alkanals; 9, 4-oxo-*trans*-2-alkenals; 10, low-molecular-mass, short-chain *n*-alkanals, particularly propanal and *n*-butanal; 11, unidentified aldehyde classification signal within the δ = 9.81-9.83 ppm region. CLO samples were prepared for ^1^H NMR analysis by the method described in section S2.1.
